# Ileocolic resection for Crohn's disease and the Kono S anastomosis: all that is gold does not glitter

**DOI:** 10.1111/ans.19181

**Published:** 2024-07-29

**Authors:** David A. Clark

**Affiliations:** ^1^ Department of Surgery Royal Brisbane and Women's Hospital Brisbane Queensland Australia; ^2^ Faculty of Medicine University of Queensland Brisbane Queensland Australia; ^3^ Department of Surgery St Vincent's Private Hospital Northside Brisbane Queensland Australia

The accumulation of evidence by which we make decisions can be slow and the accrual can create potential temporary deviations away from the final outcome as the journey continues. In this perspective is presented an important example involving ileocolic resection and Crohn's disease for consideration.

Many surgeons will have read the Luglio^1^ randomized controlled trial (RCT) from 2021 investigating the Kono S anastomosis after ileocolic resection for Crohn's disease (CD) and some will have altered their practice and will have taken up the Kono S anastomosis as their preferred anastomotic configuration. On the face of it, the findings of an endoscopic recurrence (i2 or greater) rate of 22.2% in the Kono S group *vs*. 62.8% in the stapled side to side (SSTS) (*p* < 0.001) is strong evidence of the superiority of the new technique. This coupled with the retrospective cohort studies of very low surgical recurrence out of Japanese centres that have reported five‐year surgery free rates of 95.0% in the Kono S *vs*. 81.3% in the end to end group (*p* < 0.001),^2^ make the case compelling. Certainly, it is imperative to study this anastomotic configuration rigorously, but it is important to wait for high level evidence before it is adopted as the gold standard.

It is important to remember that the primary outcome in the Luglio study was evidence of endoscopic recurrence graded at i2 (more than 5 ulcers in the ileum or anastomosis) and that this is a surrogate outcome for more important clinical outcomes such as surgical recurrence, need for new medical therapy or indeed quality of life.^3^ These long term outcomes are not yet available.

In an attempt to better predict the future disease course after an ileocolic resection, Rutgeerts developed an endoscopic score in 1984 based on a number of endoscopic findings.^3^, ^4^ This score was later modified to account for the observation that ulceration along the staple line of a SSTS may not behave the same way as ‘true’ ileal aphthous ulcers. The modified Rutgeerts score separates >5 ulcers at the staple line (i2a) and >5 ulcers in the terminal ileum (i2b). The i2b lesions have a greater likelihood of progressing to more severe endoscopic disease, and thus are considered a more important predictor of disease course.^5^

Surgical recurrence is an important outcome but is also highly subjective. The criteria for resection are not defined and of course are interpreted in light of the individual patient's symptoms and wishes. These are framed by their gastroenterologist's approaches to treatment and susceptible to temporal bias as more therapeutic options become available on the PBS. Practice in 2024 is very different to practice in 2014. This highlights the imprudence of historical cohorts as comparator groups in IBD studies.

An excellent example of temporal bias is the presentation of outcomes of the extent of mesenteric resection in ileal CD when retrospective cohorts are used. The Coffey *et al*. study of radical mesenteric resection used historical controls to conclude that a radical resection of the mesentery led to a near five‐year surgical recurrence rate of 2.9% *vs*. 40% in the conservative group (*p* = 0.003).^6^ These findings were largely repudiated this year at ECCO with the presentation of the results of the RCT of radical *vs*. conservative mesenteric resection (the SPICY trial).^7^ In this study the investigators randomized 130 patients to an extended mesenterectomy *vs*. a mesentery sparing resection. The authors found no difference in the primary outcome of endoscopic recurrence at 6 months, graded as i2b or greater (42.4% *vs*. 43.1%; *p* = 1.0). Whilst this is a surrogate outcome for clinical and surgical recurrence, it is certainly objective. The five‐year surgical outcomes are awaited.

The Luglio RCT of 74 patients now stands in stark contrast to the interim outcomes of the much larger North American RCT of the Kono S *vs*. the SSTS anastomosis.^8^ The interim results of this study were presented by Koiana Trencheva, also at the ECCO meeting this year and reported endoscopic recurrence rates of 25.9% *vs*. 27.8% (*p* = 0.775) in this interim study of the 250 patients who had reached their primary outcome of i2b or greater at post‐operative colonoscopy at 3–6 months. There are still 32 patients who haven't as yet reached their primary outcome and the final publication and long term outcomes are eagerly awaited.

The Kono S anastomosis comprises three elements that have not been independently studied. These are: 1 the supporting column; 2 the mesenteric preservation; and 3 the stricturoplasty‐like anastomotic configuration that excludes the mesentery from the anastomosis.^9^

Stefan Holubar *et al*. from the Cleveland Clinic reported on the safety of a combination of mesenteric resection with the other two elements.^10^ RCTs studying this combination are proceeding in the Cleveland Clinic, the United Kingdom and Australia.^11^

Some of the shine is certainly coming off the Kono S anastomosis. The original cohort studies have reported extraordinarily low surgical recurrence and the construct is supported by the Luglio RCT. However, larger and well‐constructed RCTs are providing conflicting evidence to cohort series. These studies need to be interpreted in the context of primary outcomes of endoscopic recurrence which are a surrogate for important and relevant clinical outcomes.

IBD patients are a heterogenous cohort and thus it is important that randomized studies are conducted to do the heavy lifting to control bias. In an ideal world, all eligible patients would be enabled to be entered into an RCT to expeditiously advance evidence but there are substantial administrative barriers to these multicentre studies.^12^ It is paramount that we investigate new operative techniques thoroughly and wait for good quality, long term evidence before we embrace these techniques as a new gold standard.

## Author contributions

**David A. Clark:** Conceptualization; writing – original draft; writing – review and editing.
